# Determinants of Non-Adherences to Long-Term Medical Therapy after Myocardial Infarction: A Cross-Sectional Study

**DOI:** 10.3390/ijerph17103585

**Published:** 2020-05-20

**Authors:** Yongwhi Park, Yong-Hwan Park, Ki-Soo Park

**Affiliations:** 1 Department of Internal Medicine, Institute of Health Sciences, College of Medicine, Gyeongsang National University, Jinju 52727, Korea; angio2000@hanmail.net; 2 Department of Internal Medicine, Gyeongsang National University Changwon Hospital, Changwon 51472, Korea; hippomac@hanmail.net; 3 Department of Cardiology, Samsung Changwon Medical Center, Changwon 51353, Korea; 4 Department of Preventive Medicine, Institute of Health Sciences, College of Medicine, Gyeongsang National University, 15-816 Jinju-daero, Jinju 52727, Korea

**Keywords:** adherence, anxiety, concern, myocardial infarction, necessity

## Abstract

Purpose: Non-adherence to medications can be classified as unintentional and intentional. The aim of this study was to establish the major determinants of each non-adherence in myocardial infarction (MI). We also evaluated the effects of non-adherences on healthy behaviors. Materials and Methods: We enrolled 510 patients >1 year after MI. Nonadherences classified as unintentional or intentional were measured by a self-reported questionnaire. Polynomial and multiple regression analysis were performed to evaluate the determinant of each type of nonadherences. Results: Among patients with nonadherence, 263 (70.7%) patients were unintentionally non-adherent while 109 (29.3%) patients were intentionally non-adherent. Psychological belief and attitude were important in unintentional non-adherence (Exp(β) = 0.917, *p* = 0.050 for anxiety; Exp(β) = 1.191, *p* = 0.001 for concerns). Beliefs about medications were the strongest determinant of intentional non-adherence (Exp(β) = 0.812, *p* < 0.001 for necessity; Exp(β) = 1.421, *p* < 0.001 for concerns). Anxiety was important determinant of intentional non-adherence (Exp(β) = 0.889, *p* = 0.015). Conclusion: Psychological factors and beliefs about medication were important determinants of both types of non-adherence. Combined approaches targeting the beliefs about medications and psychological distress are needed to improve drug adherence in patients with MI.

## 1. Introduction

Non-adherence to medication is a pandemic phenomenon found in >60% of patients taking cardiovascular medications [1]. Indeed, approximately one fourth of patients did not fill their first prescriptions after acute myocardial infarction (MI) [2]. Long-term adherence further declined over time, and only one fourth of patients remained adherent over 2 years [3]. Importantly, non-adherence to optimal cardiovascular medications was associated with a 30% higher risk of cardiovascular events in MI patients [4]. However, predicting or improving adherence to medications remains a challenge because multiple contributors are interwoven with each other in a complex way [5]. This suggests that strategies to improve adherence should be individualized and multifaceted [5,6].

The medication-taking behaviors of patients can be classified as intentional and unintentional according to the patient’s perspective [7]. Unintentional non-adherence is a passive forgetfulness to take medications, whereas intentional non-adherence is an active decision to avoid taking medications. Therefore, the major triggers and possible solutions might be divergent depending on the type of non-adherence. Unintentional non-adherence is mainly driven by socio-demographic factors associated with many practical barriers, such as age, pill-counts, and frequency of medications. On the other hand, individual psychological responses to medications are main determinants of intentional non-adherence, and therefore the elimination of such perceptual barriers may be a reasonable strategy to improving intentional non-adherence.

Patients with MI are likely to be unintentionally non-adherent to medications because of the relatively high affected age, the large number of medications needed for life-long use, and the chronicity of the disease [7]. At the same time, these patients are also vulnerable to mental illnesses, including depression or anxiety, and are susceptible to the adverse effects of medications [8]. These might increase the risk of intentional non-adherence after MI. However, little is known about the prevalence or importance of adherence patterns in MI patients. In addition, the evaluation of the different triggers of each non-adherence would be helpful to establishing the strategies for overcoming non-adherence after MI. Several studies have shown that medication concerns and negative attitudes adversely affect adherence [9,10]. Therefore, evaluating and dealing with patients’ attitudes and beliefs about drug compliance can result in a reduction in the burden of chronic disease.

The current poor understanding of contributing factors of unintentional and intentional non-adherence after MI prompted this study. This study was conducted to identify factors affecting drug compliance in patients with myocardial infarction.

## 2. Methods

Patients were eligible for the study if they have been taking optimal medications (necessarily antiplatelet was included) for at least 1 year after AMI successfully treated with percutaneous coronary intervention. The definition of MI used in this study was based on the third universal definition of MI [11]. Data collection was performed using two large cardiovascular centers located in Gyeongsangnam-do province in Korea.

The survey was conducted from January 2014 to July 2014. The interviewers were 2 well-trained nurses independent of the study. During the study period, all patients who visited the outpatient were selected as study subjects (728 patients), of which 510 maintenance myocardial infarction patients (response rate 70.0%) agreed to participate in the study. This study was approved by the institutional review board (IRB No; 2013-12-026-001) and written informed consent was obtained from all participants.

## 3. Materials

Socio-demographic data were collected by medical interviews and electronic charts, encompassing all the possible contributing factors of non-adherence, such as gender, age, educational level, monthly income, and presence of spouse. The ejection fraction on echocardiography and a number of diseased vessels on coronary angiography were used as markers of disease severity. Patients reported a dichotomous self-rated health status as either “good” or “poor”.

### 3.1. Assessment of Adherence

The participants answered three questions (yes/no) regarding unintentional non-adherence in reference to prescription medications for AMI: forget to take the prescription medication/run out of the prescription medication/careless at times about taking the prescription medication. Respondents also answered three questions regarding intentional non-adherence in reference to their medication-taking behavior over the past one month: took less medication than instructed because you felt better/worse; skipped taking medication because you felt better/worse; skipped doses of medication because you felt hassled [12].

We divided patients into the following three groups: “adherence,” in which they answered no for all six questions; “unintentional non-adherence,” in which they answered “no” for all the three intentional non-adherence questions and answered “yes” for any of the unintentional non-adherence questions; and “intentional non-adherence,” in which they answered “yes” for any of the three intentional non-adherence questions [12].

### 3.2. Beliefs about Medication Questionnaire (BMQ)

BMQ, as developed by Horne et al., was used to assess the patient beliefs (necessity/concern) regarding the medication, and BMQ has been used in other Korean studies [9,13]. Participants answered five items about necessity and five items about concern using a 5-point Likert scale (1 = strongly disagree to 5 = strongly agree). The BMQ is scored by summing the items on each subscale, ranging from 5 to 25. Higher scores on the necessity subscale indicate a stronger belief in the necessity of the medications, whereas higher scores on the concern subscale indicate that the participant has stronger concerns about taking medications.

### 3.3. Social Support

We used the ENRICHD social support instrument (ESSI) scale, which is a 7-item self-report survey, to measure the social support of the patients [14]. The questionnaire includes three questions for social support, two questions for instrumental support, the presence of a spouse, and the presence of a close contact. The response categories range from 1 (none of the time) to 5 (all of the time). Individual items are then summed for a total score, with higher scores indicating greater social support.

### 3.4. Depression and Anxiety

The symptoms of anxiety and depression were measured using the Hospital Anxiety and Depression Scale (HADS), which was developed for screening anxiety and depressive symptoms over the previous week [15].

### 3.5. Statistical Analysis

An analysis of variance was used for continuous variables, and the chi-square test was used for categorical variables. A polynomial logistic regression model was used to estimate the effects of each covariate on both the odds of unintentional non-adherence and the odds of intentional non-adherence compared with adherence, while simultaneously adjusted for demographic variables; age, sex (reference: male), spouse (reference: no), educational level (reference: elementary), monthly income (reference: <$1000) and co-morbidity (hypertension and DM). Lastly, multiple regression analysis was performed to find out factors associated with subscale (necessity, concerns) of BMQ, which are known to be related to drug adherence.

All statistical analyses were carried out using the SPSS version 25.0 software (SPSS Inc., Chicago, IL, USA).

## 4. Results

Among the 510 study participants, 372 (72.9%) were non-adherent to medications (Table 1). Unintentional non-adherence (263, 51.6%) was more prevalent than intentional non-adherence (109, 21.4%). Patients with non-adherence, whether intentional or unintentional, were found to be younger than those adherent to medications. There were no differences in socio-demographic factors between patients with medication adherence and either type of non-adherence. However, adherents had a higher prevalence of hypertension compared with non-adherents. The patients reporting poor health status were more likely to exhibit medication non-adherence, whether intentional or unintentional. Although non-adherent patients were taking a higher number of pills a day, this result had no statistical significance. The patients with intentional or unintentional non-adherence also reported a lower social support, as measured with the ESSI scale. A higher anxiety score and more concern about the medications were associated with non-adherence (Table 1).

### Factors Associated with Each Type of Non-Adherence

More pill counts (B = 0.118, *p* = 0.013) significantly increased the perceived necessity, while a good self-rated health status (B = −0.912, *p* = 0.008) attenuated it (Table 2). A poor self-rated health status (B = −0.906, *p* = 0.003) also significantly increased the concerns about medication (Table 3). Patients reporting higher anxiety symptoms (B = 0.114, *p* = 0.012) were associated with increased concerns about medication.

Patients reporting a higher anxiety score (odds ratio (OR) = 0.917, 95% CI = *p* = 0.050) were also less vulnerable to unintentional non-adherence. Worth noting, a larger concern about the medications (Exp(**β**) = 1.191, *p* = 0.001) was the most powerful predictor of unintentional non-adherence (Table 4).

Anxiety (OR = 0.889, *p* = 0.015) was an important negative predictor of intentional non-adherence, as was the case in unintentional non-adherence. Similar to unintentional non-adherence, beliefs about medication, which are necessity (Exp(**β**) = 0.812, *p* < 0.001) and concern (Exp(**β**) = 1.421, *p* < 0.001), were the strongest determinant of intentional non-adherence (Table 5).

**Figure 1:**
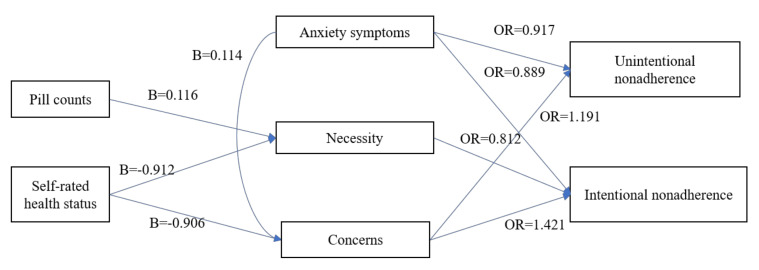
Main findings of this study.

In conclusion, anxiety symptoms and concerns about medication were associated with unintentional non-adherence, and anxiety symptoms and beliefs about medication (concerns, necessity) were associated with intentional non-adherence. In addition, belief about medication (concerns, necessity) was associated with health status, pill counts, and anxiety symptoms (Figure 1).

## 5. Discussion

The present study evaluated the determining factors of unintentional and intentional non-adherence. Non-adherence to medications was common in long-term medical therapies after MI, and unintentional non-adherence was more prevalent than intentional non-adherence. These findings were the same as in previous studies [13,16]. The novel findings of this study are as follows. (1) A high concern was the most powerful determining factor for both unintentional and intentional non-adherences. (2) Intentional non-adherence was more closely related to various psychologic factors, including beliefs about medication, as compared with socio-demographic factors. (3) Anxiety symptom was a negative predictor of both types of non-adherence. In addition, anxiety symptom had a negative effect on the perceived concerns about medications.

There are many studies related to adherence of AMI, but in general, the prescription and dosage rate of each AMI drugs were suggested. In Korea, the prescribed drugs are packaged and prepared at once, so it is not known by drug type. Recently, nearly 30% of AMI patients in Chinese cohort did not take medications as prescribed during the first month after discharge [17]. In our study, it is in the middle of the intentional nonadherence and unintentional nonadherence distributions. However, due to differences in tools, direct comparison is not possible. However, it can be seen that there are many nonadherences in AMI patients.

According to conventional concepts, unintentional non-adherence has been recognized as the product of socio-demographic factors, illness-related factors, polypharmacy, or regimen complexity [7,18,19]. However, recent studies have shown the close link between unintentional non-adherence and cognitive factors [13,16]. This suggests that tailored intervention may be needed, according to the type of non-adherence. Indeed, this study also demonstrated that age and a prior history of hypertension were associated with unintentional non-adherence but not intentional non-adherence. However, age had an inverse relationship with unintentional non-adherence. Although elderly patients were considered to be vulnerable to unintentional non-adherence, the association between age and medication adherence was not significant in previous studies [2,12,18]. This suggests that passive forgetfulness via the cognitive impairment frequently shown in elderly patients may not be a fundamental cause of medication non-adherence. The present study also discovered important findings regarding the effects of psychological distress on non-adherence. Depressive and anxiety symptoms have been recognized as important risk factors of non-adherence and cardiovascular mortality [2,20,21]. However, depressive symptom was not a predictor of either type of adherence in this study. However, anxiety and depressive symptoms were closely related to concerns about the medication, which was one of the most important determinants for both types of non-adherence.

Beliefs about medications were the most important predictor for both types of non-adherence in this study. This suggests that interventions focusing on the beliefs about medications might be the best modalities to reduce both unintentional and intentional non-adherence. However, patients with intentional non-adherence had higher concerns about the medications than those with unintentional non-adherence (14.9 ± 3.2 vs. 13.7 ± 3.9). Therefore, patients with intentional non-adherence require more carefully planned interventions to reduce concerns about medication. However, the interventions needed to improve beliefs about medications showed conflicting results [22]. This mandates an improved understanding of the individual perception of medications in order to develop effective and efficient tools for interventions. The present study showed that anxiety and depressive symptoms were associated with higher concerns regarding medications. Therefore, interventions to improve a person’s beliefs about medication should include the tools to alleviate the symptoms of anxiety and depression. We found that overall pill counts increased perceived necessity but had no association with perceived concerns. However, higher pill counts may increase the chances of forgetting to take medicines [5,7]. These conflicting results imply that patients might have an ambivalent mind toward high pill counts. If physicians ordered higher pill counts, patients might perceive more benefits from their medications but are less likely to follow them. Therefore, lower pill counts with simple regimens, such as a polypill, would be a better strategy for improving both types of non-adherence [23,24].

Management of non-adherence is difficult to attain and is rarely sustainable. Combined and multifaceted strategies targeting diverse causes of non-adherences were more effective and also time- and resource-intensive than single factor modulations [5,6]. The results of the present study corroborated the importance of perception regarding medications and psychological distress. However, efficient interventions to improve individual beliefs about medications would be difficult to achieve during busy office visits. Supplementary interventions by pharmacists and nurses also increased drug adherence, but this required considerable amounts of time and resources [5,25].

This study had some limitations. Both intentional and unintentional non-adherences were self-reported in this study, which might limit the validity of our measure. However, comparative analysis showed that self-reported measures of adherence could be considered reliable. Patient questionnaire is widely used to measure adherence to therapies in the clinical setting due to its simplicity and low cost, even though it is subject to bias of results by patients [26]. Second, there was a time gap due to the difference among questionnaires, that is, drug adherence questions over the past month, while questions of social psychological factors asked over the past week. However, most surveys tend to respond based on the latest, so there will be some degree of reliability and validity of the response. In addition, since this study included patients from only two centers, the participants included in the present study may not be representative of most MI patients in Korea, which in turn might have led to a selection bias. Because the present study only evaluated the major determinants of medication adherence, the results of the present study might not warrant the confident improvement of medication adherence. This prompts further studies on tailored interventions targeting specific types of non-adherence, as well as developing strategies to improve medication adherence and healthy behaviors at the same time.

## 6. Conclusions

Individual beliefs about medications were the most important risk factor for both unintentional and intentional non-adherences. However, socio-demographic factors have no effect on the development of intentional non-adherence. Therefore, individualized interventions to change beliefs about medications are required to improve medication adherence. Combined approaches targeting the beliefs about medications and psychological distress are needed to improve drug adherence in patients with MI.

## Figures and Tables

**Table 1:** Differences in general characteristics.

	Non-Adherence (n = 372)		Adherence (n = 138, 27.1%)	
Variables	Unintentional (n = 263, 51.6%)	Intentional (n = 109, 21.4%))		
Gender (n, %)				
Male	203 (77.2)	77 (70.6)	105 (76.1)	0.804
Female	60 (22.8)	32 (29.4)	33 (29.1)	
Age, years	62.5 ± 11.0	62.6 ± 10.8	65.7 ± 10.6	0.005
Education (n, %)				
Elementary	86 (32.7)	38 (34.9)	53 (38.4)	0.258
Middle	75 (28.5)	29 (26.6)	27 (19.6)	
High	64 (24.3)	29 (26.6)	35 (25.4)	
≥College	38 (14.4)	13 (11.9)	23 (16.7)	
Income ($/month)				0.985
≤1000	105 (39.9)	42 (38.5)	54 (39.1)	
1001–2000	70 (26.6)	31 (28.4)	39 (28.3)	
2001–3000	30 (11.4)	15 (13.8)	16 (11.6)	
>3000	58 (22.1)	21 (19.3)	29 (21.0)	
Spouse (n, %)				0.488
Yes	190 (72.2)	69 (63.3)	95 (68.8)	
No	73 (27.8)	40 (36.7)	43 (31.2)	
Self-rated health status (n, %)				0.014
Good	74 (28.1)	27 (24.8)	55 (39.9)	
Poor	189 (71.9)	82 (75.2)	83 (60.1)	
Hypertension (n, %)				0.018
Yes	107 (40.7)	46 (42.2)	71 (51.4)	
No	156 (59.3)	63 (57.8)	67 (48.6)	
DM (n, %)				0.054
Yes	56 (21.3)	26 (23.9)	32 (23.2)	
No	203 (78.7)	83 (76.1)	106 (76.8)	
Ejection fraction (n, %)				0.704
<40%	33 (12.5)	12 (11.0)	10 (7.2)	
40%–49%	67 (25.5)	28 (25.7)	37 (26.8)	
≥50%	163 (62.0)	69 (63.3)	91 (65.9)	
Diseased vessel (n, %)				0.274
1	106 (40.3)	47 (43.1)	48 (34.8)	
2	85 (32.3)	39 (35.8)	50 (36.2)	
3	72 (27.4)	23 (21.1)	40 (29.0)	
Pill counts, mean ± SD	8.2 ± 3.4	8.6 ± 4.0	7.7 ± 3.6	0.203
ESSI, mean ± SD	23.0 ± 6.0	22.2 ± 5.8	23.9 ± 6.1	0.028
HADS (Anxiety) mean ± SD	22.4 ± 2.9	21.8 ± 4.0	23.4 ± 3.0	0.001
HADS (Depression) mean ± SD	9.5 ± 3.5	9.8 ± 3.5	10.1 ± 3.3	0.068
BMQ (Necessity) mean ± SD	17.4 ± 3.3	16.9 ± 4.1	17.6 ± 3.2	0.140
BMQ (Concerns) mean ± SD	13.7 ± 2.9	14.9 ± 3.2	12.7 ± 2.5	<0.001

DM = diabetes mellitus, ESSI = ENRICHD social support instrument, HADS = Hospital Anxiety and Depression Scale, BMQ = belief about medication.

**Table 2:** Factors related to belief about medication (necessity).

	Necessity		
Variables	B	95% C.I. for B	*p* value
**Pill counts**	**0.116**	**0.025~0.207**	**0.013**
Ejection fraction (reference: < 40%)			
40%–49%	−0.744	−1.855~0.367	0.189
≥50%	−0.766	−1.790~0.258	0.142
**Self-rated health status (Good/Poor)**	**−0.912**	**−1.586~−0.238**	**0.008**
ESSI	0.052	−0.006~0.109	0.077
Anxiety symptom	0.054	−0.049~0.156	0.303
Depression symptoms	0.072	−0.032~0.176	0.175

S.E. = standard error, C.I. = confidence interval, DM = diabetes mellitus, ESSI = ENRICHD social support instrument. Adjusted for age, sex, spouse, educational level, monthly income and co-morbidity (hypertension and DM).

**Table 3:** Factors related to belief about medication (concerns).

	Concerns		
Variables	B	95% C.I. for B	*p* value
Pill counts	0.031	−0.049~0.110	0.447
Ejection fraction (reference: < 40%)			
40%–49%	−0.002	−0.967~0.964	0.997
≥50%	−0.462	−1.352~0.428	0.308
**Self-rated health status (Good/Poor)**	**−0.906**	**−1.493~−0.320**	**0.003**
ESSI	0.000	−0.050~0.049	0.984
**Anxiety symptom**	**0.114**	**0.026~0.203**	**0.012**
Depression symptoms	0.089	−0.002~0.179	0.054

S.E. = standard error, C.I. = confidence interval, DM = diabetes mellitus, ESSI = ENRICHD social support instrument. Adjusted for age, sex, spouse, educational level, monthly income and co-morbidity (hypertension and DM).

**Table 4:** Determining factors of unintentional non-adherence.

	Unintentional Non–Adherence (Reference: Adherence)		
Variables	OR	95% C.I.	*p* value
Pill counts	1.061	0.986–1.142	0.116
Ejection fraction (reference: < 40%)			
40%–49%	0.498	0.201–1.235	0.133
≥50%	0.539	0.233–1.248	0.116
Self-rated health status (Good/Poor)	0.713	0.437–1.165	0.177
ESSI	1.003	0.962–1.046	0.882
**Anxiety** symptom	**0.917**	**0.842–1.000**	**0.050**
Depression symptoms	0.938	0.967–1.015	0.111
Necessity	0.924	0.852–1.002	0.055
**Concerns**	**1.191**	**1.079–1.316**	**0.001**

OR = odds ratio, C.I. = confidential interval, DM = diabetes mellitus, ESSI = ENRICHD social support instrument. Adjusted for age, sex, spouse, educational level, monthly income and co-morbidity (hypertension and DM).

**Table 5:** Determining factors of intentional non-adherence.

	Intentional Non-Adherence (Reference: Adherence)		
Variables	OR	95% C.I.	*p* value
Pill counts	1.084	0.985–1.192	0.099
Ejection fraction (reference: < 40%)			
40%–49%	0.546	0.162–1.844	0.330
≥50%	0.611	0.196–1.902	0.395
Self-rated health status (Good/Poor)	0.673	0.339–1.335	0.257
ESSI	0.995	0.941–1.052	0.869
**Anxiety** symptom	**0.889**	**0.808–0.977**	**0.015**
Depression symptoms	1.000	0.907–1.103	0.999
**Necessity**	**0.812**	**0.729–0.904**	**<0.001**
**Concerns**	**1.421**	**1.245–1.623**	**<0.001**

OR = odds ratio, C.I. = confidential interval, DM = diabetes mellitus, ESSI = ENRICHD social support instrument. Adjusted for age, sex, spouse, educational level, monthly income and co-morbidity (hypertension and DM).

## Abbreviations

AMI	Acute myocardial infarction
BMQ	Belief about medication questionnaire
ESSI	ENRICHD social support instrument
HADS	Hospital Anxiety and Depression Scale
N-C	Necessity minus concerns,
DM	Diabetes mellitus
C.I	Confidential interval
